# Potential zoonotic spillover at the human–animal interface: A mini-review

**DOI:** 10.14202/vetworld.2024.289-302

**Published:** 2024-02-07

**Authors:** Ima Fauziah, Herjuno Ari Nugroho, Nova Dilla Yanthi, Rida Tiffarent, Sugiyono Saputra

**Affiliations:** Research Center for Applied Microbiology, Research Organization for Life Sciences and Environment, National Research and Innovation Agency (BRIN), KST Soekarno, Jalan Raya Jakarta Bogor Km 46 Cibinong, Bogor, West Java, Indonesia

**Keywords:** avian influenza, COVID-19, emerging infectious diseases, public health, wildlife market

## Abstract

Wildlife markets and wet wildlife markets, a type of human–animal interface, are commonly trading centers for wild-caught and captive-exotic animals as well as their products. These markets provide an ideal environment for spillovers of zoonotic and emerging infectious diseases (EIDs). These conditions may raise serious concerns, particularly in relation to wildlife species that frequently interact with humans and domestic animals. EIDs pose a significant risk to humans, ecosystems, and public health, as demonstrated by the current COVID-19 pandemic, and other previous outbreaks, including the highly pathogenic avian influenza H5N1. Even though it seems appears impossible to eliminate EIDs, we may still be able to minimalize the risks and take several measures to prevent new EIDs originated from animals. The aim of this study was to review several types of human–animal interfaces with a high risk of zoonotic spillover, infectious agents, and animal hosts or reservoirs. Identifying those factors will support the development of interventions and effective disease control in human–animal interface settings.

## Introduction

The human–animal interface is a phrase used to explain how humans and animals might come into contact and provide a path for the spread of infectious disease agents, such as viruses and bacteria, by direct or indirect contact through contaminated environments or inanimate objects [1, 2]. Wildlife and wet wildlife markets are among the human–animal interface types that have the potential for spillover and transmission between humans and animals, causing zoonotic diseases [3]. Zoonotic diseases are infectious diseases caused by various pathogenic agents, such as bacteria, parasites, fungi, viruses, and prions, that are naturally transmitted between vertebrate mammals and humans [4]. Most emerging infectious diseases (EID) outbreaks are caused by zoonotic diseases (60.3%), with wildlife pathogens contributing to 71.8% of all zoonotic diseases [5, 6].

Nearly all recent pandemics involve exposure to certain animal species with direct contact with humans, increasing the risk of disease transmission [6–8]. Over 200 animal-associated human infections, infestations, and zoonoses have been identified, of which 35% (70/200) are associated with exotic pets [9, 10]. Wildlife is a significant source of human diseases, accounting for approximately 75% of emerging human infections worldwide [9]. A survey of more than 1400 human diseases found that 61% potentially originated from zoonotic sources [11, 12]. However, the importance of zoonoses from pets should not be downplayed as they can also be deadly [13].

Transmission of infectious diseases between wild and domestic animals is becoming increasingly important [14]. There is still a lack of scientific knowledge about how the vast majority of infectious agents are transmitted. Animals may be exposed to livestock diseases, which may have serious consequences for their populations. In addition, many EIDs, including zoonotic diseases, have been traced back to wildlife [15, 16]. Pathogens without intermediate stages, such as viruses, bacteria, or protozoa, are the most crucial emerging wildlife pathogens. RNA viruses with high mutational rates are prime candidates for emergence [17].

In this review, we aimed to summarize several types of human–animal interfaces with a high risk of zoonotic spillover, infectious agents, and their animal hosts or reservoirs. Characterizing those aspects as well as the role of wildlife trade as a significant source of EIDs is crucial to emphasize the critical public health risks associated with wildlife in human–animal interfaces that may lead to the future pandemics.

## Potential Spillover in Wildlife Markets and Wildlife Wet Markets

Wildlife markets and wildlife wet markets vary from small stalls to large multi-storey complexes with hundreds of vendors selling domesticated and captive-exotic species, although mostly deal in wild-caught animals [18–20]. In the wildlife-pet trade, birds are the most commonly traded species, followed by reptiles and mammals [21]. Depending on the region and country [22, 23], these markets differ significantly in terms of their characteristics and the types of species offered. Wildlife markets typically trade live animals for pets, whereas wet wildlife markets trade animals as meat for consumption [8, 24].

Several wildlife-related activities raise public concern, including wildlife hunting, which threatens wildlife extinction due to uncontrolled or poorly regulated animal trade, and animal processing and handling, such as animal slaughter [3, 25, 26]. However, it remains unclear whether human exposure to live animals on wildlife markets is proportionate in certain aspects. Contamination variables related to the handling of potentially infectious animals and their derivatives in wet markets are more intense but shorter-term, whereas they are less intense but longer-term in wildlife markets [8, 27]. In contrast, pets obtained from wildlife markets have become part of the family or as companion animals, leading to increased contact and exposure of potential pathogens to humans. Meanwhile, market operators or the traders regularly deal with a wide range of animals, their derivatives, cages, and related materials and interact with the visiting public which lead to widespread interactions and provides multiple opportunities for cross-contamination [27].

Wildlife markets or wildlife wet markets provide opportunities for EIDs as various animal species from different origins, ecosystems, and taxa are crammed together in cages, sharing unclean and unnatural conditions, diets, water sources, and disease vectors [3, 8, 28]. For instance, defecation of animals will facilitate the exchange of pathogenic microorganisms and force interactions of various species that would not normally occur [9, 28]. EIDs are more common in areas with a higher population density, increased livestock production, increased agricultural activities, and where appropriate, a higher biodiversity, particularly among mammals. Tropical regions with high biodiversity, extensive tropical forests, and severely damaged habitats are also more susceptible to EIDs [9, 29].

Interspecies and intraspecies mixing of animals in wildlife markets can promote the shedding and recombination of pathogens, especially viruses, within new hosts, thereby increasing pathogenicity among animals and humans [30]. Captive animals often experience elevated stress levels due to high housing densities, new environments, or exposure to unfamiliar species, further weakening their immune system responses and increasing EIDs transmission and virulence [29, 31, 32]. This concern is more pronounced in the markets for sheltered wild species, which tend to respond more strongly related to transportation or market conditions than domesticated animals. The density of animal species in wildlife markets plays an important role in the spread of EIDs. High animal densities can facilitate the transmission of diseases within species, between species, and between animals and humans [5, 33, 34], depending on the market structure and the proximity of animals to each other. Higher animal densities increase the likelihood of interspecies mixing and subsequent cross-contamination, leading to disease transmission, adaptation, and subsequent zoonotic development [35, 36].

Numerous potentially pathogenic agents have been discovered in the intestines and tissues of both wild and captive animals [37], indicating that human microbial pathogens and microparasites could infest animals from various locations and sources [3]. A study of fecal samples from many species of hoofed mammals, primates, carnivores, ratites, and reptiles found that 45% contained zoonotic intestinal parasites [37]. Many potential human pathogenic microorganisms and parasites are inherently normal and commensal to other animal species and are practically non-eradicable [9]. For example, commensal *Salmonella* bacteria in prey mice can invade snake intestines and it potentially transmit through fecal-oral route to humans [38]. Regardless of whether the animals are caught or reared in captivity, the pathogen reservoir remains a key factor. The transmission of diseases from animals to humans may occur through proximity, consumption, and handling of animals or their products, as animals are susceptible to different diseases. Once a pathogenic agent has been introduced into the human population, it can spread from humans to humans, leading to outbreaks [24].

## Wildlife and Notable Viral Zoonotic Diseases

According to a study, at least 138 viral infections in humans originated in pet animals [12]. On the other hand, along with the trend of human populations moving closer to previously isolated wildlife populations, the complex interplay of viral adaptations between wild species, domesticated species, and humans may facilitate the emergence of new zoonotic diseases. Due to its increased virulence, rapid spread, and inadequate medical knowledge or treatment, this disease can be dangerous to humans [8].

Significant outbreaks of vector-borne diseases have affected humans and non-human animals. For example, West Nile virus (*Flaviviridae*) that cause more than 15,000 human deaths was also discovered in wild reptiles in the United States [39]. Numerous potential viral pathogens can spread from invertebrates to humans through the predator-prey food chain [3]. Various non-commensal and novel human infections can be introduced to wild-caught predators that consume different prey, along with their microbiome and virome [3]. The Ebola virus outbreak that widespread in several countries in Africa is associated with wildlife hunting and trading of contaminated wildlife meat [40]. Ebola virus is able to survive in that, allowing them to endure storage, transport, and husbandry conditions [12], which leads to possible global spread transmission of pathogens to other areas [9, 41, 42]. According to WHO, Ebola cases was not observed in Asian countries, however, Ebola-like virus detected with very low proportion in fruit bats in China and Bangladesh but not in Thailand [43].

Wild animals in wildlife markets may also contaminate pets and other animals, such as chickens, dogs, cats, and rabbits, and serve as intermediate reservoirs [3]. Small wild mammals such as bats and rodents are among the most important known natural hosts of zoonotic viruses [44]. A wide range of viruses, including SARS-CoV, Middle-East respiratory syndrome coronavirus (MERS-CoV), SARS-CoV-2, henipaviruses, and filoviruses, are associated with bat viruses and cause severe epidemic or endemic diseases in humans [45, 46]. Approximately 66,000 people are estimated to be silently infected with unidentified bat coronaviruses (CoVs) per year [47]. Bats have a significant diversity of CoVs, picornaviruses, astroviruses, and a potentially novel *Bornaviridae* genus [48]. In addition to SARS-related CoV-2 and HKU4-CoV-like (University of Hong Kong 4 Coronavirus-like) viruses, picornaviruses and respiroviruses are likely to circulate between bats and pangolins [46, 48]. Bats also serve as reservoir hosts for several zoonotic viruses, such as Hendra and Nipah, which were developed in Australia and East Asia, respectively [49, 50]. Rodents are also recognized as hosts of numerous human infections, such as Hantaviruses and Mammarenaviruses, as well as human CoVs OC43 and HKU1, which significantly impact public health [51, 52]. Between 2015 and 2022, among the 27 families of mammalian viruses there were eight viruses that successfully identified and characterized, highlighting their pathogenicity originating from 1981 wild animals and 194 zoo animals in South China [48]. In addition, potential cross-species transmission of RNA viruses (paramyxovirus and astrovirus) and DNA viruses (parvovirus, porcine circovirus 2, porcine circovirus 3, and pseudorabies virus) between wildlife and domestic animals has been discovered [48].

Recent epidemics and pandemics, such as COVID-19, avian influenza, swine influenza, monkeypox, Ebola, and MERS, are examples of diseases that are transmitted from animals to humans [3]. The highly pathogenic avian influenza A (H5N1) virus is highly contagious and occurs when humans have direct or close contact with infected birds [53]. Therefore, visiting or mixing animals in live poultry markets is an important risk factor for infection [54]. RNA viruses, such as the avian influenza virus (AIV) and CoVs, are known for their tendency to undergo variations due to frequent replication errors caused by mutation, deletion, rearrangement, and recombination, providing them with enormous opportunities for adaptation and evolution [6, 7]. Many aspects of the nature of these infections and the origin of some outbreaks remain unknown [24].

## Swine Influenza Virus, Animal Reservoirs, and Pandemic Potential

H1N1 swine influenza, commonly known as swine flu, is a common respiratory disease caused by influenza A virus (IAV) in pigs worldwide. Swine flu is an important economic disease for the swine industry as it can cross-species barriers to infect humans and cause significant economic losses for pig producers [55]. Swine flu infection is sporadic in humans and usually results from exposure to infected pigs in live markets or pork industry [3, 56]. It was first isolated from pigs in the 1930s and has been the predominant swine influenza strain for the next 60 years [57, 58]. In 1918, a deadly influenza pandemic was caused by the H1N1 influenza virus, also known as the Spanish flu, which is a progenitor of swine flu variants [58, 59]. Direct transmission of the virus from pigs to humans is rare, with only 12 documented cases in the United States since 2005 [60]. Pigs may harbor influenza virus strains; potentially reinfecting humans after immunity has weakened, posing a risk of cross-species transmission due to genetic variation [59, 60].

In 2009, a new strain of H1N1 swine flu spread rapidly across the world, leading the World Health Organization (WHO) to declare it as a pandemic [55]. The 2009 H1N1 virus spread rapidly worldwide among humans and resulted in 43 to 89 million cases and 1799 deaths in 178 countries worldwide [57, 58]. However, the 2009 H1N1 virus was not zoonotic because it did not originate from pigs. Instead, the virus is transmitted through airborne droplets between humans and potentially through contact with contaminated surfaces, which could transfer the virus to the eyes or nose [57, 58]. Influenza A pandemics, exemplified by 1918 and 2009, can arise when the transmission of influenza virus is highly efficient [60, 61]. Subsequently, in 2015, a mutated H1N1 virus was responsible for the spread of the 2009 pandemic in India [60]. Swine influenza, which is often linked with other swine pathogens, contributes to the porcine respiratory disease complex, causing increased mortality and economic losses [62]. In spite of its name, swine flu cannot be acquired by consumption of pig products. Two-way transmission of IAVs between humans and swine has also been recorded, with the 2009 H1N1 virus exemplifying two-way transmission [61]. Despite the large number of swine influenza vaccines used in the swine industry, swine influenza remains an important economic disease for the swine industry and cannot be efficiently controlled [55].

## Monkeypox Virus (MPXV), Animal Reservoirs, and Pandemic Potential

Monkeypox is a rare viral disease transmitted between animals and humans, caused by an enveloped double-stranded DNA virus of the *Poxviridae* family [63]. MPXV was first recognized in 1959 during outbreaks of macaque monkeys in Denmark [64]. MPXV has a broad host range, from rodents, and rope squirrels, to sooty mangabeys, suggesting potential zoonotic circulation in the wild [63]. In the 1970s, the number of smallpox cases increased due to the cessation of smallpox vaccination. The first case was reported in a 9-month-old child in the Democratic Republic of the Congo in 1970 [65]. Subsequent cases have been reported in Nigeria and other West and Central African regions, particularly in remote areas [66]. The 2022 outbreak marked by the WHO as a significant global concern showed an unusual concentration in Europe and the Americas with 71,096 cases by October 7, 2022 [67].

The majority of cases, notably in non-endemic regions such as the U.S., Brazil, and Spain, displayed a significant male predominance, particularly among men aged 31–40 years and those who have sex with men [68]. Investigations have traced the outbreak in North America and Europe to sources such as the pet trade and travel, with interesting reports suggesting possible sexual transmission [65]. Europe and the Americas, in particular 14 countries, accounted for more than 90% of the cases, whereas only 345 cases were reported in seven African countries with historical endemicity [67]. Asia and Oceania reported fewer cases, and Iran reported its first case in August 2022 [67]. Monkeypox, sometimes transmitted between humans, is a challenge due to multiple modes of transmission, such as inhalation, skin contact, and bodily fluids [65]. In spite of increased outbreaks linked to factors such as the cessation of smallpox vaccination and animal consumption, there is no definitive cure [65]. The WHO classifies monkeypox as a re-emerging disease with potential for bioweapons, emphasizing the need to be vigilant, especially in individuals with underlying health conditions [67].

## Avian Influenza Viru (AIV), Animal Reservoirs, and Pandemic Potential

The AIV has the potential to cause significant outbreaks in both domestic and wild bird populations and poses a risk to humans who come into contact with infected animals. AIV belongs to the *Orthomyxoviridae* family and is categorized as an A-type influenza virus with single-stranded RNA [69]. IAVs are important pathogens that affect human and animal health. Migratory wild birds, especially waterfowl, serve as natural hosts and reservoirs of AIV [70]. In addition, AIV were able to cross the species barrier and infect mammals such as rats, mice, weasels, ferrets, pigs, cats, tigers, dogs, and horses [71].

The primary wild species involved in AIV transmission cycle include waterfowls, gulls, and shorebirds [71]. Direct interaction between these wild bird species and farmed birds is a likely route for the virus to spread [72]. Wild birds can carry various avian influenza virus strains within their respiratory or intestinal tract [73]. Wild birds play a crucial role in the evolution and maintenance of AIV, particularly during low seasons [70, 71].

Within avian species, 16 hemagglutinin (HA) subtypes and nine neuraminidase subtypes of IAVs have been identified [74]. Since 1918, H1N1, H2N2, and H3N2 subtype viruses have caused four influenza pandemics. Both H1N1 and H3N2 viruses continue to cocirculate globally among human populations [75, 76]. Importantly, various subtypes of avian influenza viruses are known to circulate among animals and have sporadically crossed into human populations, with some demonstrating pandemic potential [77, 78].

Although several subtypes of avian influenza viruses have been detected in domestic birds, particularly those in contact with wild avian species, only three HA subtypes, namely, H5, H7, and H9 have been known to be transmitted to and detected in domestic bird populations [70]. Notably, some strains with HA genes from H5 or H7 subtypes exhibited highly pathogenic avian influenza (HPAI) characteristics, resulting in significant challenges for the global poultry industry [69, 74].

Over the past century, outbreaks of AIV caused by distinct H5 subtypes have occurred in eight nations or regions [71, 79, 80]. In 1959, the H5N1 virus led to the first outbreak of HPAI in chickens in Scotland. In 1966, H5N9 was responsible for the first AIV outbreak in turkeys in Canada. From 1983 to 1985, H5N2 caused a number of epidemics in chickens and turkeys in the United States. In 1983, H5N8 caused outbreaks in chickens, turkeys, and ducks in Ireland. In 1991, H5N1 caused an outbreak in turkeys in England. From 1994 to 1995, H5N2 caused a number of outbreaks in chickens and turkeys in Mexico [79]. In 1997, H5N1 and H5N2 viruses caused outbreaks in chickens in Hong Kong [73] and Italy [80], respectively.

**Figure-1:**
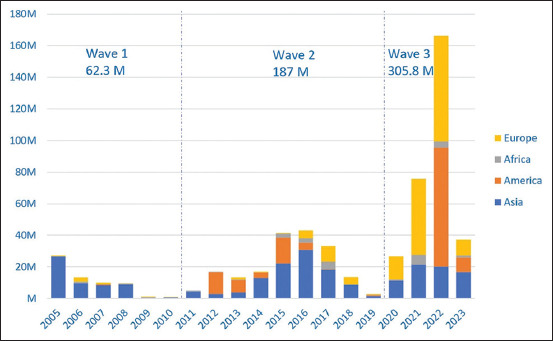
The number of cases, culled, and died of poultry and non-poultry caused by highly pathogenic avian influenza H5 since 2005 in Asia, America, Africa, and Europe reported in the OIE-World Animal Health Information System [83].

In 2002, the first H5N1 outbreak occurred in Hong Kong [81]. Subsequently, several Asian countries, including Vietnam, Thailand, Indonesia, China, Japan, South Korea, Cambodia, and Laos, experienced H5N1 outbreaks in 2003 and 2004 [81, 82]. There is no information on the scale of poultry losses during outbreaks before 2004. From January 2005 to June 2023, HPAI caused by H5 subtypes resulted in the loss of a staggering 555.1 million poultry and non-poultry animals worldwide (Figure-1), as reported in OIE-World Animal Health Information System (OIE-WAHIS) [83]. These outbreaks spread across Asia, Africa, Europe, and the Americas in three waves.

The first wave (2005–2010), mainly caused by H5N1, resulted in the culling and death of 62.3 million poultry and non-poultry animals. Most of these epidemics are concentrated in Asian countries, followed by European and African countries [83]. The second wave (2011–2019) affected Asia, Europe, Africa, and the Americas and was caused by various H5 virus subtypes. It resulted in the culling and death of 187 million poultry and non-poultry animals [83]. The ongoing third wave, which starts in 2020, is mainly due to H5N8 and H5N1 viruses. In particular, outbreaks have been reported in the Americas, Europe, and Asia, and some incidents have been documented in African countries. As of the end of June 2023, this wave led to the culling and death of 305.8 million poultry and non-poultry animals. H5N1 viruses accounted for 204 million of the 389 million poultry losses, and H5N8 viruses accounted for 111 million; other H5 subtypes contributed to the remaining 74 million poultry losses [83]. According to OIE-WAHIS [83], there is a seasonal pattern for HPAI, with the lowest spread occurring in September, rising in October, and reaching its peak in February. A significant number of cases, culling, and deaths have been observed in the third wave across the Americas, Europe, and Asia in a relatively short period, highlighting the potential severity of the ongoing wave if disease control measures remain unchanged [83].

In 1997, H5N1 was transmitted from birds to humans in Hong Kong, which resulted in the infection of 18 people, six of whom died tragically. This is the first documented case of human infection with the lethal H5N1 virus, which has drawn widespread attention [84]. According to data reported by the WHO [85], 874 cases of human infection with avian influenza A (H5N1) virus have been recorded across 23 countries between January 2003 and April 2023 (Table-1). Of these 874 cases, 458 proved fatal, resulting in a case fatality rate of 52%. China and Laos have reported 75 cases of H5N6 virus infection, while Russia has reported seven cases of H5N8 virus infection [85]. Between 2003 and April 2022, 947 human cases related to various AIVs were reported, of which 488 cases resulted in deaths [74].

**Table-1:** Avian influenza cases in human caused by H5N1 around the world according to the WHO [85].

Country	2003-2010		2011-2019		2020-April 2023		Total	
			
	Cases	Death	Cases	Death	Cases	Death	Cases	Death
Azerbaijan	8	5	0	0	0	0	8	5
Bangladesh	1	0	7	1	0	0	8	1
Cambodia	10	8	46	29	2	1	58	38
Canada	0	0	1	1	0	0	1	1
Chile	0	0	0	0	1	0	1	0
China	40	26	13	5	2	1	55	32
Djibouti	1	0	0	0	0	0	1	0
Ecuador	0	0	0	0	1	0	1	0
Egypt	119	40	240	80	0	0	359	120
India	0	0	0	0	1	1	1	1
Indonesia	171	141	29	27	0	0	200	168
Iraq	3	2	0	0	0	0	3	2
Lao	2	2	0	0	1	0	3	2
Myanmar	1	0	0	0	0	0	1	0
Nepal	0	0	1	1	0	0	1	1
Nigeria	1	1	0	0	0	0	1	1
Pakistan	3	1	0	0	0	0	3	1
Spain	0	0	0	0	2	0	2	0
Thailand	25	17	0	0	0	0	25	17
Turkey	12	4	0	0	0	0	12	4
UK	0	0	0	0	1	0	1	0
US	0	0	0	0	1	0	1	0
Viet Nam	119	59	8	5	1	0	128	64
Total	516	306	345	149	13	3	874	458

H7 HPAI subtypes have caused outbreaks of poultry disease worldwide. According to data from OIE-WAHIS [83], different H7 HPAI viruses caused 106 outbreaks and caused the loss of almost 33 million poultry worldwide from January 2005 to November 2022. The H7N3 virus caused 77 outbreaks in North America, which resulted in the deaths of over 29 million birds. In addition, H7N7 viruses caused 10 outbreaks in Europe and South Korea, while H7N9 viruses caused outbreaks in the United States and China. In Australia, outbreaks have been caused by at least three types of H7 viruses. These findings highlight the ongoing threat of H7 virus to the global poultry industry [84, 86, 87].

H7 HPAI and low pathogenic avian influenza have historically caused human infections, with a total of 1687 human cases documented across eight countries between 1959 and 2019 [84, 86, 87]. In 2003, 89 human cases of H7N7 HPAI virus infection were reported in the Netherlands, which resulted in the unfortunate death of a veterinarian [88]. In China, one human case of H7N4 virus infection and 1568 human cases of H7N9 virus infection have been reported, with 616 of the H7N9 virus infections proving fatal [89]. Many human cases associated with H5 and H7 virus infections highlight the high susceptibility of humans to these avian influenza strains. Typically, human infections are caused by exposure to virus-infected birds or contaminated environments, with limited cases of human-to-human transmission [90]. Effective animal control measures are necessary to prevent these viruses from entering human populations and acquire the capacity for human-to-human transmission.

## Coronaviruses (CoVs), Animal Reservoirs, and Pandemic Potential

**Figure-2:**
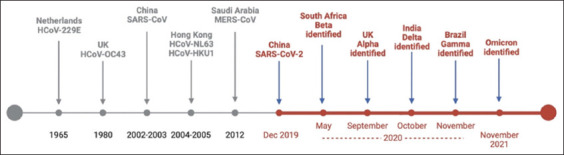
The discoveries of human coronaviruses and variants of Severe acute respiratory syndrome coronavirus-2 in chronological order [Source: Panel derived from Timeline by BioRender.com (2023), retrieved from https://app.biorender.com/biorender-templates/figures].

CoVs have been detected in various bird species and mammals, including bats, rodents, camels, cows, pigs, dogs, palm civets (*Paguma larvata*), raccoon dogs (*Nyctereutes procyonoides*), and pangolins [28, 91, 92]. Until the emergence of SARS-CoV at the end of 2002, CoVs were mainly associated with mild illness similar to seasonal flu [91]. More than 300 CoVs have been identified, but only seven Human coronaviruses (HCoV) are known to cause diseases in humans. Among these four subtypes, two alpha (HCoV-229E and NL63) and two beta (HCoV-OC43 and HKU1) generally induce mild-to-moderate symptoms similar to those of the common cold [93]. Between 1965 and 1980, HCoV-229E and HCoV-OC43 were responsible for 10%–15% of common cold cases, primarily occurring in winter [94]. HCoV-NL63 was isolated in 2004 from a child with bronchitis in the Netherlands [95], whereas HCoV-HKU1 was isolated in 2005 from a Hong Kong patient with chronic pulmonary disease [96]. The remaining three beta coronavirus subtypes have been associated with severe diseases: SARS-CoV, responsible for the 2002 outbreak; MERS-CoV, responsible for the 2012 outbreak; and SARS-CoV-2, which led to COVID-19, the ongoing pandemic originating in Wuhan, China in December 2019 (Figure-2) [7, 91].

According to the WHO [85], between 2002 and 2003, an outbreak of SARS-CoV from Civet cats and bats in Guangdong, China, resulted in 8096 infections and 774 deaths, with a fatality rate of 9.6%. MERS-CoV, which first emerged in Saudi Arabia in 2012, resulted in 2502 confirmed cases and 861 deaths, with a fatality rate of 34.4% [85]. The COVID-19 pandemic reported by the WHO [85] has witnessed more than 768 million confirmed cases and 6,952,522 deaths worldwide from late December 2019 to July 2023. Europe (275,777,982) and the USA (2,958,446) reported the highest number of confirmed cases and deaths, respectively. The United States of America experienced the most significant impact of the COVID-19 pandemic, with over 103 million confirmed cases and 1.1 million deaths (Table-2).

**Table-2:** The countries with the highest number of cases and deaths caused by COVID-19 worldwide according to the WHO [85].

Country	Cases	Country	Deaths
US	103.436.829	US	1.127.152
China	99.298.516	Brazil	704.488
India	44.995.332	India	531.915
France	38.997.490	Russia	399.814
Germany	38.437.756	Mexico	334.336
Brazil	37.704.598	UK	228.286
Japan	33.803.572	Peru	221.261
South Korea	32.866.350	Italy	190.987
Italy	25.908.353	Germany	174.979
UK	24.647.430	France	167.985
Russia	22.974.289	Indonesia	161.879
Turkey	17.004.677	Iran	146.306
Spain	13.980.340	Colombia	142.922
Vietnam	11.621.626	Argentina	130.476
Australia	11.531.080	Spain	121.852

SARS-CoV-2 has a number of mutations that lead to the emergence of various variants. The WHO and other health organizations classify these variants on the basis of their characteristics and implications for public health [85, 97, 98]. Variants of concern (VOC), variants of interest (VOI), variants of high consequence, and variants under monitoring (VUM) or variants being monitored (VBM) are classified. The WHO classification of SARS-CoV-2 variants is detailed in Table-3. VOI, according to the WHO, is variants with genetic mutations likely to impact virus characteristics, such as transmissibility, virulence, antibody evasion, treatment susceptibility, and detectability [85]. It may also show a growth advantage compared to other circulating variants, leading to increased prevalence and epidemiological concerns. The variant shall be classified as a VOC if it meets the criteria of a VOI and, on the basis of a risk assessment carried out by the WHO Technical Advisory Group on SARS-CoV-2 Virus Evolution, has a significant impact on the capacity of health systems, results in more severe clinical diseases, and substantially reduces the effectiveness of current vaccines [85]. Many variants initially classified as VOCs were later reclassified as VOIs or VBMs because they were previously circulating VOCs or VOIs [97, 98]. The WHO and the Centers for Disease Control and Prevention (United States and the European Union) regularly update the working definitions for VOC, VOI, VBM, and formerly monitored variants [85, 97, 98].

**Table-3:** SARS-CoV-2 variants classifications by the WHO [85].

WHO label	Pango lineage	GISAID clade	First detection	Date of designated
Variants of Concern				
Alpha	B.1.1.7	GRY	UK, September 2020	VOC: December 18, 2020 PVOC: March 09, 2022
Beta	B.1.351	GH/501Y.V2	South Africa, May 2020	VOC: December 18, 2020 PVOC: March 09, 2022
Gamma	P. 1	GR/501Y.V3	Brazil, November 2020	VOC: January 11, 2021 PVOC: March, 09, 2022
Delta	B.1.617.2	G/478K.V1	India, October 2020	VOI: April 04, 2021 VOC: May 11, 2021 PVOC: June 07, 2022
Omicron parent lineage	B.1.1.529	GR/484A	Multiple countries, November 2021	VUM: November 24, 2021 VOC: November 26, 2021 PVOC: March 14, 2023
Variants of interest				
Epsilon	B.1.427 B.1.429	GH/452R.V1	US, March 2020	VOI: March 05, 2021 PVOI: July 06, 2021
Zeta	P. 2	GR/484K.V2	Brazil, April 2020	VOI: March 17, 2021 PVOI: July 06, 2021
Eta	B.1.525	G/484K.V3	Multiple countries, December 2020	VOI: March 17, 2021 PVOI: September 20, 2021
Theta	P. 3	GR/1092K.V1	Philippines, January 2021	VOI: March 24, 2021 PVOI: July 06, 2021
Iota	B.1.526	GH/253G.V1	US, November-2020	VOI: March 24, 2021 PVOI: September 20, 2021
Kappa	B.1.617.1	G/452R.V3	India, October 2020	VOI: April 04, 2021 PVOI: September 20, 2021
Lambda	C.37	GR/452Q.V1	Peru, December 2020	VOI: June 14, 2021 PVOI: March 09, 2022
Mu	B.1.621	GH	Colombia, January 2021	VOI: August 30, 2021 PVOI: March 09, 2022

PVOC=Previous variants of concerns, PVOI=Previous variants of interest, VOC=Variants of concern

Alpha- and beta-variants of SARS-CoV-2 are characterized by higher transmissibility and increased resistance to antibody neutralization. It is noteworthy that the alpha-variant was found to be 100 times more deadly than the original SARS-CoV-2 strain [99]. On 21 September 2021, the WHO [85] designated the Alpha variant as a “VUM.” However, after the emergence of Delta variants, the global circulation of Alpha variant significantly declined in 2022, likely due to its impact on vaccine-induced immunity [85, 97].

The Delta variant, first discovered in India in October 2020, rapidly spread to the United Kingdom and other parts of the world by mid-April [100, 101]. Infection caused by Delta variant was associated with a higher likelihood of hospitalization compared to Alpha variant, indicating increased virulence [101,102]. According to reports, Delta variant is approximately 60% more transmissible and lethal than Alpha variant and remains the dominant strain globally until October 2022 [100]. When first identified in South Africa in November 2021, WHO_immediately classified the Omicron variant as a “VOC” [85]. Omicron variant stands out due to its extensive mutation profile and heightened contagiousness, leading to a rapid surge in infections in South Africa [103, 104]. It quickly spread to several countries and emerged as one of the most dominant variants by December 2021, giving rise to multiple lineages, each with distinct genetic mutation patterns [85, 105–107].

Despite its high transmissibility, the Omicron variant generally leads to milder disease than the delta- and alpha-variants [85]. In November 2020, the beta-variant (B.1.351) was first identified in South Africa, and the gamma-variant (P.1) emerged in Brazil [108, 109]. In addition, two Epsilon lineages (B.1.427 and B.1.429) were discovered in California, displaying increased transmissibility, infectivity, and severity compared to earlier variants and lineages [110, 111]. The Lambda variant (lineage C.37), initially detected in Peru in August 2020 and recognized as a VOI by the WHO on June 14, 2021, is known to be more resistant to neutralizing antibodies than other variants [112]. There is a suspicion that it may exhibit reduced vaccine efficacy compared to the alpha- and gamma-variants [113].

Bats are widespread worldwide, particularly in tropical regions, and are one of the most diverse and numerous groups of animals, second only to rodents [28, 114]. Among bats, the Rhinolophus genus is a known natural reservoir of SARS-like viruses, providing strong evidence that SARS-CoV has a wildlife origin [28, 49]. In 2013, researchers discovered a coronavirus known as sarbecovirus RaTG13 in a horseshoe bat species (*Rhinolophus affinis*) in Yunnan province [91]. However, sarbecovirus RaTG13 cannot infect human cells due to incompatibility between its spike (S) protein and human cell membrane receptors (angiotensin-converting enzyme 2) [49, 79, 115]. Although SARS-CoV-2 shares 96.2% genetic similarity with bat coronavirus RaTG13, there is currently no evidence supporting the hypothesis that SARS-CoV-2 was transmitted directly from bats to humans [92, 115]. Experimental studies have shown that not all bat species can sustain SARS-CoV-2 replication [115].

Although bats have been considered potential carriers of CoVs (including SARS-CoV and MERS-CoV) due to the recent emergence of SARS-CoV-2 [92], studies have indicated that the zoonotic risk of viral transmission is relatively uniform among various bird and mammal species, with bats not posing a notably higher risk than other wildlife [92, 116]. Pangolins are another wildlife species that may harbor coronaviruses. Multiple SARS-CoV-2-related viruses were detected in Malayan pangolin tissues imported from South-east Asia to Southern China between 2017 and 2019 [117, 118]. There is no conclusive evidence that bats or any other wild animal is a direct source of SARS-CoV-2 in humans [115, 117].

Predicting which viruses have the potential to cause significant human outbreaks is challenging, given the diversity of viruses present and the dynamic recombination processes occurring among lineages within the bat reservoir [28, 91]. It is crucial not to blame bats or any other animal for the current epidemic [28]. Preliminary findings from experimental infections conducted in various countries suggest that SARS-CoV-2 can infect a range of animals, including pigs, ferrets, minks, catarrhine primates, cats, dogs, and tigers [28, 119–122]. However, no conclusive evidence exists that these animals can transmit the virus to humans [123]. Given the extensive global spread of COVID-19 with millions of human infections, determining whether a wild animal harboring SARS-CoV-2 serves as a natural reservoir host of the virus or is simply another victim of the ongoing pandemic presents a significant challenge [28, 123].

## One Health and Action Plans

Monitoring and evaluating microbial ecology can help us understand the complex interplay between sustainable development, human health, and microorganisms [124]. One Health has emerged as a systematic approach to global health security [125]. There are different levels of legal regulation in wildlife markets worldwide. In China, wildlife markets are illegal, and enforcement is uneven. After the COVID-19 outbreak, China enhanced monitoring and enforcement procedures to maintain the ban effectively [8]. In North America (Canada and the United States), the regulation of wildlife markets is different [3]. Cultural markets based on traditional overseas culinary practices are common but are often subject to limited public health oversight, despite some efforts to improve animal welfare and public health standards [27]. In Europe (e.g., the Netherlands, Germany, Spain, and Czech Republic), wildlife markets are generally permissible, but the sale of protected species is prohibited [3, 25].

Animals sold in wildlife markets can be either wild-caught or captive-bred within their respective countries, with sales occurring at local, national, or global levels [18–20, 23, 25, 126, 127]. The standards of animal welfare in these markets range from inadequate to highly abusive. Studies have shown that this treatment compromises the immune systems of animals, making them more susceptible to infection and contributing to pathogen shedding [128–130]. As a result, the presence of diverse animal species in close confinement facilitates the exchange of commensal, opportunistic, and pathogenic microorganisms, creating numerous opportunities for infectious agent spillover. This increases the risk of epidemics and pandemics originating from different sources [12, 29, 39, 131].

It is often difficult to distinguish between animals caught in the wild and those bred in captivity. However, correctly identifying captive-bred and locally sourced animals before their interaction with animals of unknown origin and health status is crucial to minimize contamination and infection risks, thereby enhancing biosecurity and epidemiological traceability [132, 133]. These dynamics conclude that numerous handling, transportation, and disturbance events that occur frequently in wildlife markets are important for animal welfare and the potential for a variety of wildlife-associated pathogens, which should be appropriately considered in all animals regardless of the apparent source and endpoint sale circumstances [3].

Modern transport facilitates a rapid global movement of many people who may be stressed, immunocompromised, or ill and may carry pathogens. This makes it possible to distribute animals around the world within a very short time after capture, handling, and storage [9, 12, 41, 131]. In addition, wildlife markets and related trade centers frequently exist in areas with high human population densities, encouraging the rapid spread of pathogenic pathogens [3, 9]. According to human population and disease models, EIDs are more likely to occur in more densely populated areas with a more diverse range of wildlife [29]. Protective distances between diseases and human populations are continuously and systematically closed by human behavior and practices [134]. As has already been pointed out, major animal and public health outbreaks, such as avian influenza, monkeypox, swine flu, SARS-CoV, and most recently COVID-19, are linked to wildlife markets as a likely source of origin. Additional new CoVs, ebolaviruses, and hantaviruses are other potential sources of outbreaks from wildlife markets [24, 135].

The commercial exploitation of wildlife biodiversity, the inhumane treatment of animals, the role of modern globalized transportation in hastening the spread of pathogens from remote to domestic areas, and the risks associated with emerging diseases linked to wildlife trade and conditions in wet/wildlife markets [9, 29, 39, 42, 136] have been continuously reported. One Health is committed to achieving optimal human and environmental health and aims to combine research disciplines, sectors, and public health organizations to improve a comprehensive understanding of the linkages between humans, animals, plants, microbes, and ecosystems as a single integrated system [125]. This study focuses on the role of microbial dynamics in socioecological systems at the local, regional, and global levels.

The One Health model is a collaborative platform; however, a fundamental integration of biomedical science, global ecology, and sustainability is still needed. Although ecological and evolutionary sciences are sometimes dismissed as unrelated to human health, they are critical for understanding disease emergence and risk [12, 137]. As the cost of DNA sequencing continues to decrease, the screening of wild animal populations for viral composition, evolution, and dynamics can be more effective before animal viruses pose a threat to human populations [138]. Collaborative research teams must monitor and comprehend the dynamics of pathogenic agents in natural ecosystems and assess pandemic risk within the context of global ecology and sustainable development [139]. Therefore, “one health, one welfare, and one biology” emphasizes that the health and welfare of humans, animals, and the environment are closely related [4, 22, 39, 135, 137].

## Conclusion

This review underlines the critical importance of wildlife and potential zoonotic disease surveillance in our rapidly changing world, driven by escalating human impact on natural ecosystems and increased interactions between diverse species, which elevate the risk of disease transmission. Our comprehensive analysis of wildlife markets as reservoirs of EIDs, especially viral EIDs, as well as their potential transmission pathways to humans and domestic animals, highlights the serious public health risks. To effectively mitigate these risks and protect global health, biodiversity conservation should be prioritized, the surveillance of wildlife viruses should be intensified, and collaboration between international, national, and local actors should be strengthened. To address the complex challenges posed by emerging and recurrent diseases, it is necessary to adopt a holistic “One Health” approach, which recognizes the interdependence of human, animal, and environmental health. Increased public awareness of the importance of wildlife research indicates the need for proactive action, and our integrated system of surveillance of wildlife diseases at international, national, and local levels is crucial for identifying the origin of the disease and implementing control measures. This coordinated effort reduces disease transmission risk and strengthens global biosecurity. There is an urgent need to expand the surveillance of wildlife-borne viruses, particularly at the wildlife-domestic animal-human interface, to prevent outbreaks of emerging and emerging diseases. The combination of these elements highlights the urgency of resolving wildlife-related infectious diseases and stresses the need for a collaborative and multifaceted approach to protecting the health of our planet and its inhabitants.

## Author’s Contributions

IF and SS: Conceptualized and designed the study and drafted the manuscript. IF, HAN, NDY, RT, and SS: Collected the literature, edited the manuscript, and finalized the manuscript. All authors read, reviewed, and approved the final manuscript.
